# Editorial: Physiology and Physiopathology of Breath-Holding Activity

**DOI:** 10.3389/fphys.2022.858371

**Published:** 2022-02-17

**Authors:** Frédéric Lemaître, François Billaut, Fabrice Joulia

**Affiliations:** ^1^CETAPS EA 3832, Faculty of Sports Sciences, University of Rouen, Rouen, France; ^2^CRIOBE UAR 3278, CNRS-EPHE-UPVD, Papetoai, French Polynesia; ^3^Department of Kinesiology, Laval University, Quebec, QC, Canada; ^4^C2VN, INRAE 1260, INSERM 1263, Aix Marseille Université, Marseille, France; ^5^UFRSTAPS, Université de Toulon, Toulon, France

**Keywords:** hypoxia, cardiovascular, pulmonary, training, sport

Research into voluntary apnea is becoming increasingly popular in varied laboratories and in the field around the world. In the last 10 years, as many articles have been published than between 1954 and 2011. Breath-hold diving is truly an antique practice; for example, Alexandre Legrand employed professional breath hold divers called “Urinator” in his army, and Japanese Ama and Philippine Bajau people have been known to dive for hundreds of years to gather food. More recently, apnea diving became a high-level competitive sport with impressive performances (e.g., world record for static apnea of 11 mins 35 s and for depth No-Limit diving −214 m), but although constant progress is made, human performance is still far from the prowess of marine mammals (e.g., *Ziphius cavirostris*, apnea duration: 137 mins and 2,992 m depths). Despite major anatomical and physiological differences between humans and marine mammals, humans present with an interesting physiological response to apnea to limit the effects of hypoxia and/or pressure increase, in which the main physiological functions such as pulmonary, cardiovascular, and nervous functions can be modulated acutely and chronically. The Research Topic focuses on these varied facets but also their trainability and their consequences on health in relation to some hypoxia-related pathologies such as sleep apnea syndrome, pulmonary edema and decompression sickness.

## The Research Topic Articles

Freedivers (Tetzlaff et al.) and spearfishermen (Diniz et al., 2014) present an increase in total lung capacity and a decrease in residual volume because of their specific ventilatory training. This allows them to increase their performance in duration and depth, pushing back the physiological breaking point and favouriting the equalization (McCulloch et al.). Apnea training is therefore a relevant hypoxia training for several physical activities since it induces hypoxia and hypercapnia despite a low intensity of exercise (Joulia et al., 2003; Lemaître et al., 2009; Guimard et al.; Pla et al.) Grossman et al. reported how swimmers should better manage their ventilation per arm cycle during aerobic events to avoid consuming too much oxygen (Grossman et al.). Moreover, Guimard et al. demonstrated that performing dynamic apnea at 30% of maximal aerobic power is the best compromise between exercise intensity and apnea duration to induce a sufficient diving reflex in non-apnea trained participants. In fact, as long as the exercise intensity is mild, the diving reflex can overwhelm the muscle metaboreflex (Di Giacomo et al.). Dynamic apnea can therefore be used in other physical activities to induce hypercapnia, hypoxia and increased lactatemia even during low intensity efforts (Guimard et al.). During static and dynamic apneas, the dive reflex decreases oxygen consumption through bradycardia and peripheral vasoconstriction, and preserves oxygen-dependent organs through increased cerebral and cardiac perfusion (Joulia et al., 2009; Elia et al., 2021). The diving reflex is accentuated with face immersion, but there is no significant difference when apnea was performed only with the face immersed (dry condition) or the total body immersed (Nordine et al.). Those adaptive responses have been described in both spearfishermen and freedivers, and are correlated with a high adenosine release which exerts powerful bradycardia and vasodilation in coronary and cerebral blood vessels (Marlinge et al.). Moreover, an increase in serum heat shock protein levels in response to higher nitric oxide levels could improve cardiovascular adaptation to hypoxia in elite freedivers (Solich-Talanda et al.). Despite a low production of free radicals observed in freedivers during exercise and dynamic apnea (Joulia et al., 2002), it seems that there are no genetic predispositions to limit the oxidative stress in freedivers (Cialoni et al.). Joulia et al. (2002) had previously shown that the blood lactate concentrations recorded during dynamic apneas were lower in freedivers compared to non-divers despite the same exercise intensity. In addition, it has been shown that this increase in lactate concentration are accentuated when apneas were performed with face immersion (Bouten et al.). Despite diving reflex allows the freedivers to be more economical with oxygen, they become hypoxic depending on the duration of apnea and the intensity of exercise performed during dynamic apneas. These hypoxias can induce arrhythmias in both humans and marine mammals (Kjeld et al.; Costalat et al., 2021). Even if these cardiac rhythm disorders are reversed after the end of apnea their long-term effects remained unknown. Repetitive dives in apnea are not without risk since they can contribute to an increase in nitrogen dissolution in the tissues and then favouriting decompression sickness (Kohshi et al.). Interestingly, for freedivers, decompression illness is poorly understood and unlike in scuba diving, it is only cerebral in nature (Kohshi et al.). This suggests different mechanisms may occur within the spinal cord or skin (Edge and Wilmshurst, 2021) or in distal arteries (Arieli, 2021). It is also possible that a pulmonary shunt is created artificially due to the movement of blood during diving which increases the pulmonary artery pressure (Kohshi et al.). Both deep and repetitive breath-hold diving can lead to endothelial dysfunction (Eichhorn et al., 2017) that may play an important role in the genesis of neurological decompression sickness (Barak et al., 2020). The freediver “narcosis” has also recently been hypothesized but is still very poorly understood today (Tetzlaff et al.). The mechanisms put forward appear to favor a mechanical hypothesis via increases in cerebral blood flow and disturbances in cerebral autoregulation as described in elite freedivers (Moir et al., 2019). Depth induces haemoptysis in freedivers (Barković et al., 2021) and increases the extravascular lung water (Boussuges et al., 2011). Some freedivers present a genetic predisposition to develop haemoptysis during breath hold diving (Cialoni et al., 2015). Haemoptysis affects up to 25% of freedivers during championships, and in addition to its physiopathology of barotraumatic origin, this phenomenon may reduce oxygenation and increase the risk of syncope. Its characterization and prevention are therefore essential for the safety of freedivers. Beyond all these concepts, the use of apnea as a physiological model also appears relevant in the context of other pathologies such as, for example, sleep apnoea (Taylor et al.). Taylor et al. tested the hypothesis that individuals with documented obstructive sleep apnea (OSA) exhibit greater muscle sympathetic nerve discharge and synchronous blood oxygen level-dependent signal responses during volitional simulation of central or OSA by Mueller Maneuvers (MM) and apneas. The hemodynamic and neural responses to both apneas and MMs were in fact essentially identical in participants with and without OSA, indicating that recurring episodes of cyclical apnea during sleep does not alter cortical or peripheral neural responsiveness.

## Conclusions

Past research on apnea as well as the recent advances published in the current Research Topic really highlight breath-holding as an excellent model for studying the interactions between key physiological functions. It allows understanding how our body responds and adjusts to different chemical (*via* chemoreceptors) and mechanical (*via* the baroreflex) stimuli to maintain its homeostasis. Breath-holding is also an excellent stimulus to generate natural hypoxia. This poikilocapnic hypoxia may be incorporated into training periodization to prepare the organism for the lack of oxygen during a trip to altitude or simply to improve performance at sea level. Apnea may also be considered as preconditioning stimulus to better withstand health-damaging hypoxic situations, for example during a heart attack or stroke. Future studies are warranted in this area to refine the exercise characteristics and hypoxic dose of such apneic training for achieving the desired physiological outcome.

## Author Contributions

FL, FB, and FJ wrote together the editorial. All authors contributed to the article and approved the submitted version.

## Conflict of Interest

The authors declare that the research was conducted in the absence of any commercial or financial relationships that could be construed as a potential conflict of interest.

## Publisher's Note

All claims expressed in this article are solely those of the authors and do not necessarily represent those of their affiliated organizations, or those of the publisher, the editors and the reviewers. Any product that may be evaluated in this article, or claim that may be made by its manufacturer, is not guaranteed or endorsed by the publisher.
